# Neglected Tarlov cysts: a case of a Tarlov cyst with spermatorrhea

**DOI:** 10.1186/s40001-021-00514-w

**Published:** 2021-05-08

**Authors:** Pan Sun, Wangbing Xu, Yongxiang Ye, Faming Zhong, Xuan Wan, Yong Li

**Affiliations:** 1 Academy of Integrative Medicine, Fujian University of Traditional Chinese Medicine, Fuzhou, 350122 Fujian People’s Republic of China; 2 Spinal Department of Orthopedics, Affiliated Hospital of Jiangxi University of Traditional Chinese Medicine, 445, Bayi Avenue, Donghu, Nanchang, 330004 Jiangxi People’s Republic of China

**Keywords:** Tarlov cyst, Spermatorrhea, Low back pain, Misdiagnosis

## Abstract

**Background:**

Tarlov cysts are a commonly misdiagnosed condition, which can present with many rare symptoms. We report a case of a Tarlov cyst with spermatorrhea and review the pertinent literature.

**Case presentation:**

A 42-year-old male patient had a history of spermatorrhea for > 10 years, but was incorrectly diagnosed as the patient and the doctors consistently mistook the symptoms for a genitourinary disease. Magnetic resonance imaging showed that two cysts in the sacral canal. The diagnosis was Tarlov cyst. We performed surgery to remove the cyst and the symptoms of spermatorrhea disappeared after the operation.

**Conclusions:**

This case demonstrates that orthopedics and urologists should improve their understanding of Tarlov cysts to avoid misdiagnosis and mistreatment.

## Background

Tarlov cysts (TC) are a common neurosurgical condition, in which patients are mostly asymptomatic or display mild symptoms [1]. Some TCs are large enough to compress the adjacent nerve root and cause symptoms, such as low back pain, numbness, weakness in the lower extremities, incontinence or difficulty controlling urination and defecation, and sexual dysfunction. However, TCs with spermatorrhea as the symptom are very rare. We report a case of a male patient with a TC, which resulted in spermatorrhea for > 10 years.

## Case description

**Fig. 1:**
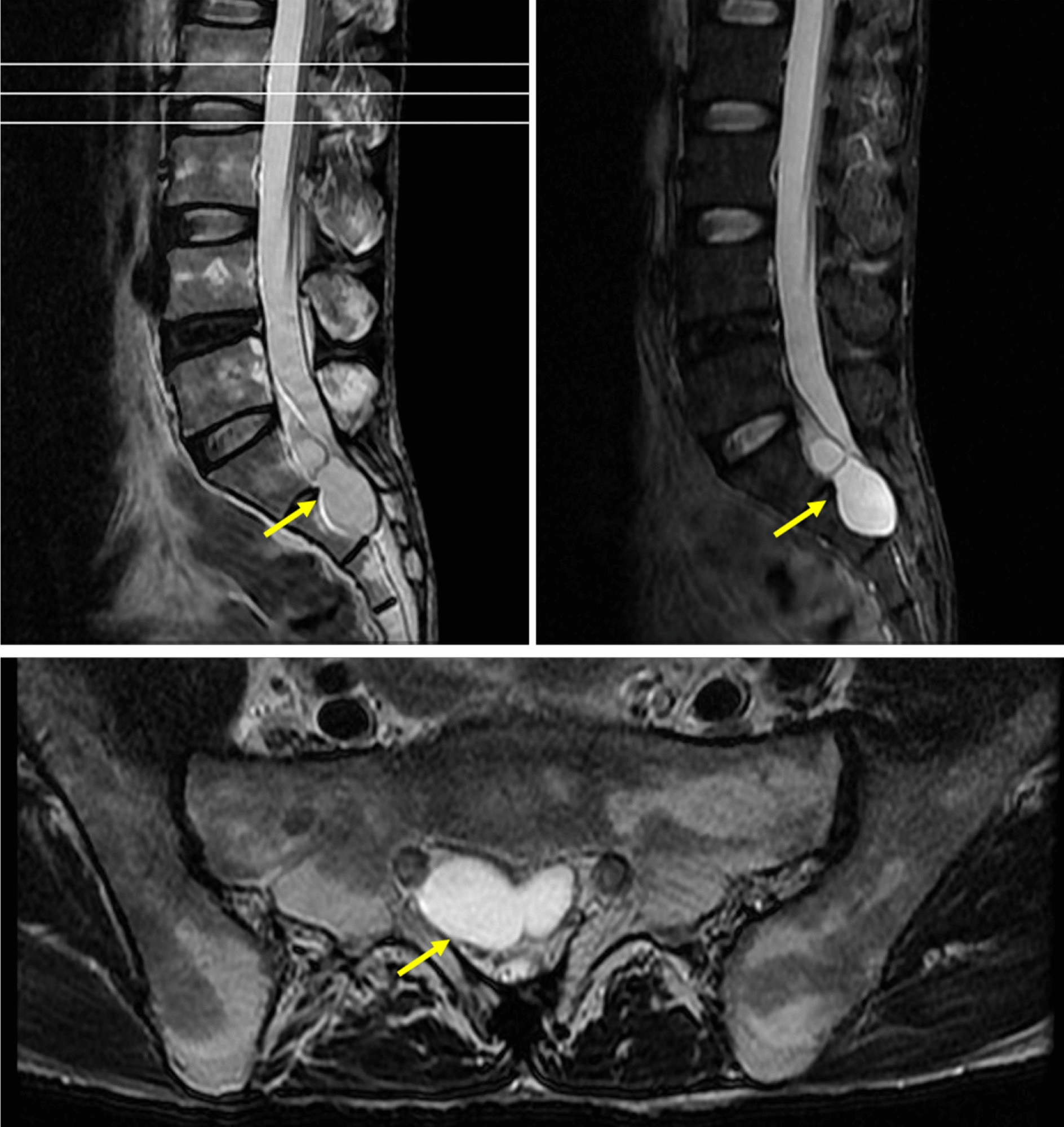
Preoperative lumbar spine MRI. T2 signal shadows in the S1–2 spinal canal

A 42-year-old male was admitted to the Acupuncture and Moxibustion Department of Affiliated Hospital of Jiangxi University of Traditional Chinese Medicine for low back pain accompanied by numbness and weakness of lower limbs for half a year. After acupuncture and moxibustion treatment failed to demonstrate significant results, the patient was transferred to The Second Orthopedics Department. The patient also complained of a history of nightly spermatorrhea that lasted for > 10 years and experienced urine incontinence, meeting the diagnosis for pudendal numbness. The patient had been treated in the Urology Department of several hospitals, but with poor results. After an MRI of the lumbar spine, we identified the potential cause: two quasicircular T2 signal shadows were observed in the S1–2 sacral canal, the larger one was ~ 2.0 × 2.8 cm in size (Fig. 1). Thus, we decided to surgically remove the TC.

## Surgical technique

**Fig. 2:**
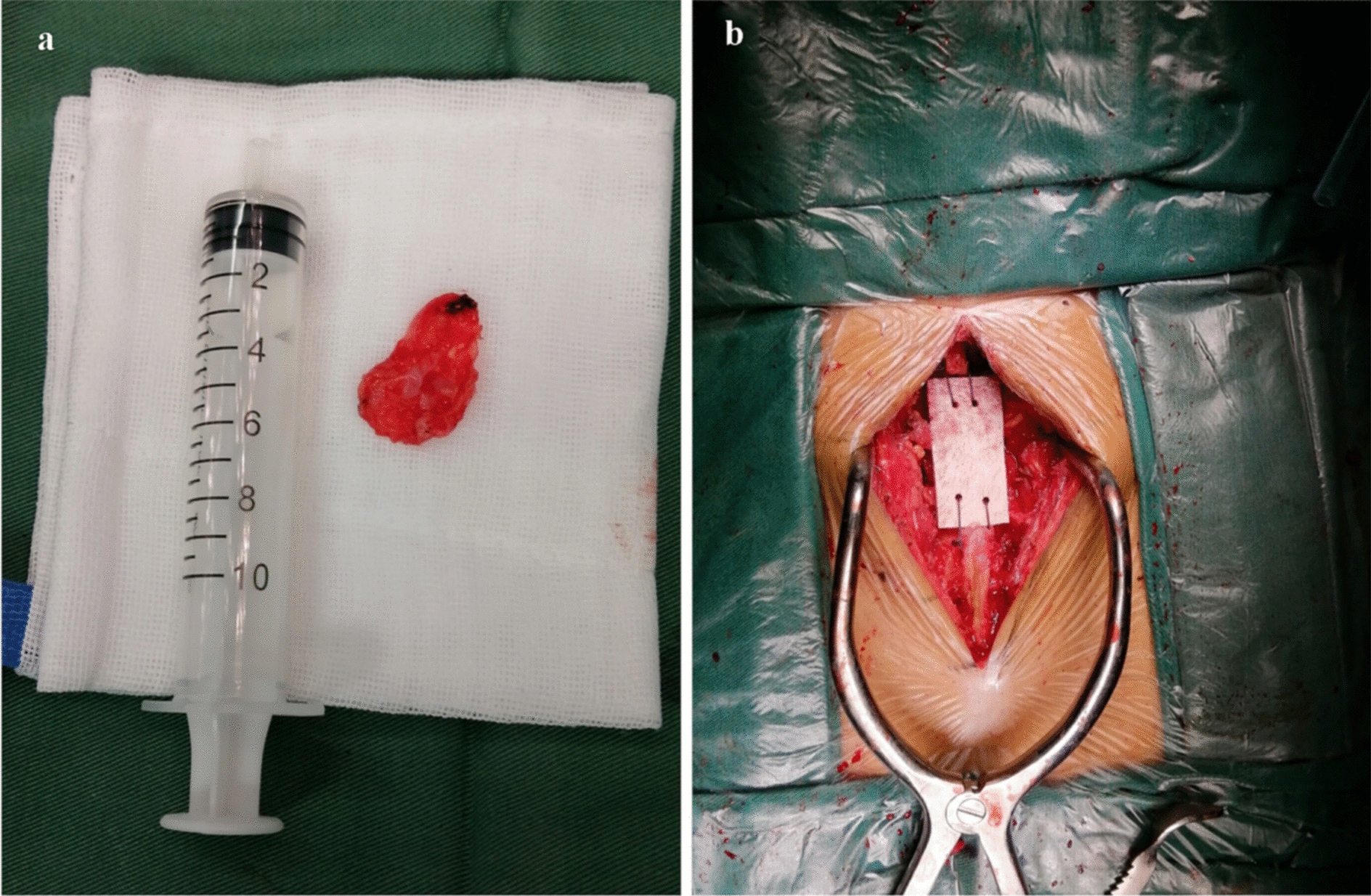
**a** Tarlov cyst was isolated during surgery. **b** Allograft bone was placed at the vertebral lamina

The vertebral plate of S1 and S2 was opened to expose the cyst, the cyst was connected to the dural sac, and there was no nerve root inside the cyst. The cyst was trimmed and shaped under the microscope. Sharp removal of the cyst with microscissors (Fig. 2a) was performed to avoid damage to nerve roots during excision; blunt dissection is not permitted. The cyst connection hole was identified and closed. The allograft bone was placed at the vertebral lamina (Fig. 2b).

**Fig. 3:**
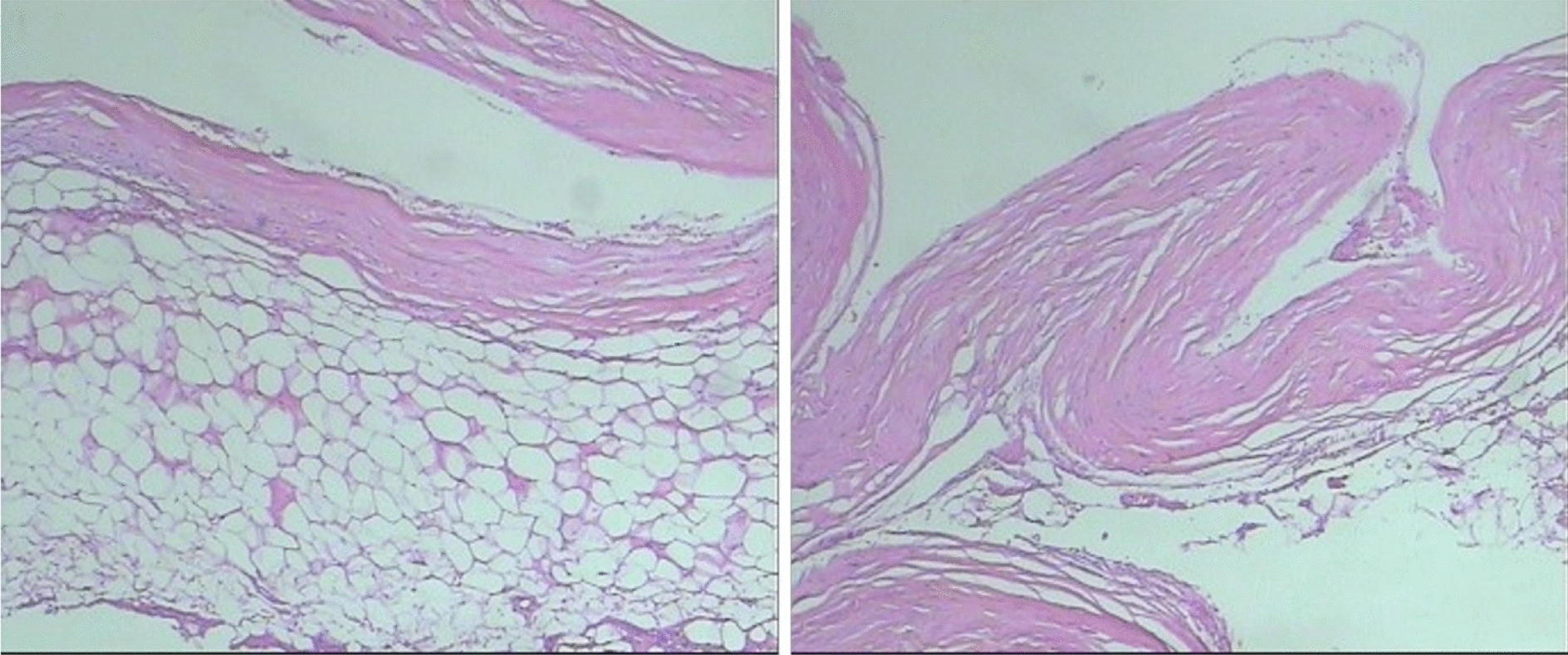
Pathological diagnosis identified cyst wall fibrous tissue hyperplasia, which was consistent with clinical diagnosis

**Fig. 4:**
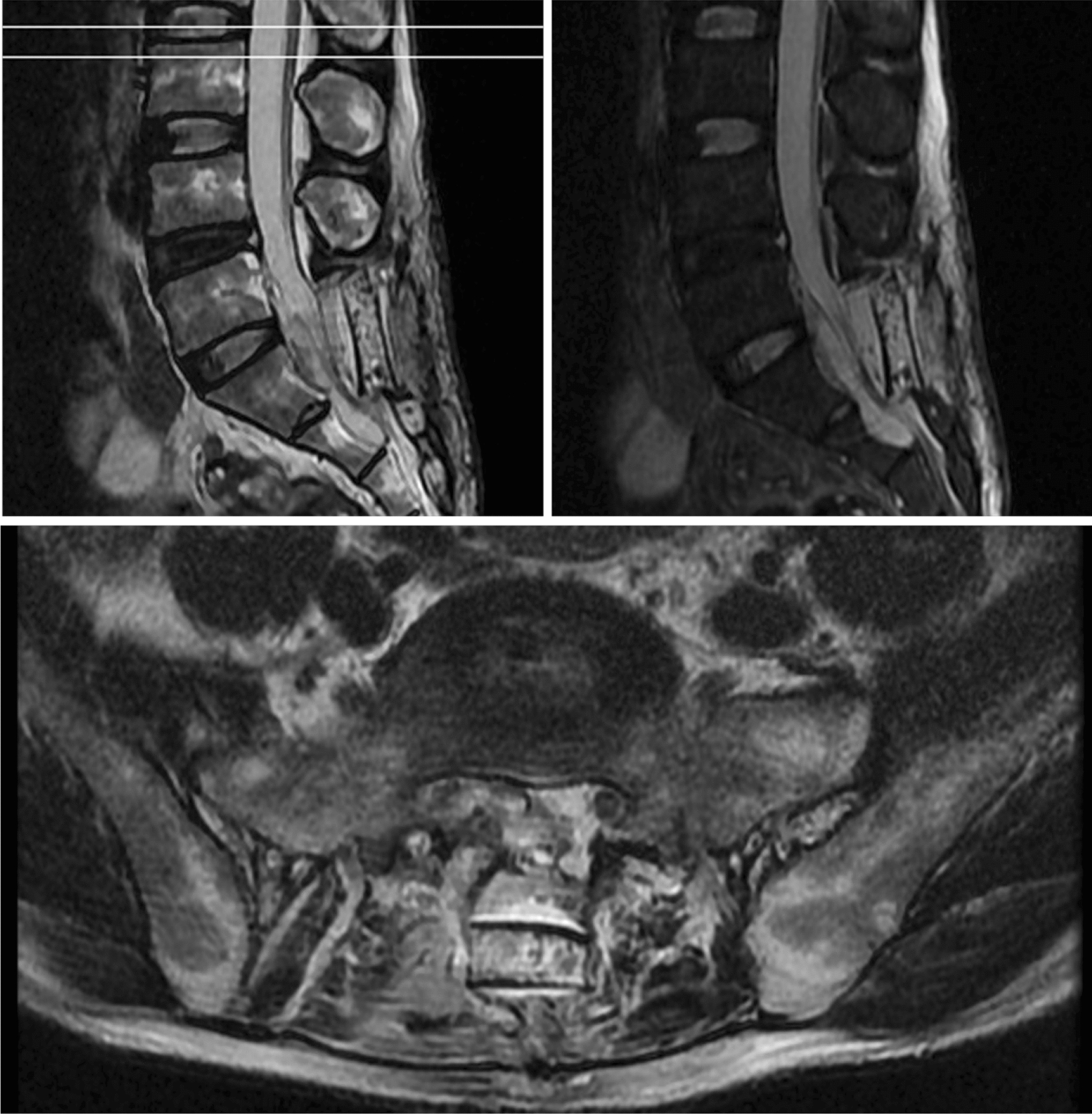
Postoperative lumbar spine MRI. S1–2 spinous process (see fixator shadow), presenting postoperative changes

After surgery, the patient’s condition was stable, and he could gradually get out of bed after 1 week. The pathological examination indicated: no clear lining epithelium, fibrous tissue hyperplasia with vitreous degeneration in the cyst wall and fatty tissue around the cyst wall (Fig. 3). MRI of the lumbar spine identified: an S1–2 spinous process (see fixator shadow), presenting postoperative changes, and a posterior margin of a S1–2 nodular long T2 signal shadow, which was ~ 1.6 × 2.1 × 2.4 cm (Fig. 4). Then, 3 months after the surgery, no symptoms of spermatorrhea appeared, and the numbness of lower limbs and urine incontinence improved significantly. The long-term efficacy requires further follow-up.

## Discussion and conclusions

TC is a spinal cord disease that had gained increased attention in recent years. Patients may present with chronic pain in the sacrococcygeal region or with decreased sensory muscle strength in both legs, decreased Achilles reflexes, abnormal perineal sensations, urinary incontinence (bladder dysfunction) and constipation (rectal dysfunction) [2]. Lumbar spine MRI is required to rule out TC patients who visit the clinic with complaints of the aforementioned symptoms. The vast majority of TCs are asymptomatic, but ~ 1% of patients will present with symptoms [3]. If conservative treatment is ineffective after the definitive diagnosis, surgical treatment should be performed to avoid lasting nerve damage [2, 4]. Therefore, TCs must be correctly diagnosed. However, diagnosis of TCs in clinical practice remains difficult, and patients can present with rare symptoms. For instance, TCs have been reported to cause abdominal pain, dyspareunia, vaginal neuralgia and depression in some patients [4–7]. To the best of our knowledge, the present study was the first report of a case of TC with spermatorrhea as the primary symptom. Due to these rare symptoms, it is difficult for doctors to determine a correct diagnosis, and the common causes of misdiagnosis have been summarized here from previous literature.

1. Lack of specificity of symptoms: clinical symptoms of TCs are mainly caused by compression of the sacral plexus by the cyst. These may be asymptomatic or non-specific in the early stages. As the cyst grows larger and the pressure becomes progressively worse, neurological symptoms appear [8]. Patients with TCs often experience back and leg pain, as well as burning pain in the perineum, numbness in the lower limbs, sensory disturbances, abnormal urination and defecation, and sexual dysfunction. The symptoms are varied and can lead to misdiagnosis [9–12]. In the present case, the patient’s main symptom was spermatorrhea. However, the condition was mistakenly considered to be a genitourinary disease, and the patient sought long-term medical treatment with little success, until after half a year when the patient developed back pain, as well as numbness and weakness in both legs. The diagnosis was not clear until examination at the Orthopedic Department.
2. Limitations on diagnostic thinking: urologists may overlook nerve symptoms, including pain and numbness in the legs, while orthopedists may overlook symptoms such as abnormal urination and sexual dysfunction. Moreover, doctors may not collect a detailed patient history. The limitations in diagnosis may be because clinicians lack a comprehensive understanding of TCs, and may first consider only the common and frequently occurring diseases, failing to further analyze the symptoms that do not meet the diagnosis criteria [9].
3. Incorrect or inadequate imaging tests: TCs are often associated with lumbosacral pain, but lumbosacral X-rays rarely demonstrate positive findings. Furthermore, TCs may only be found in the presence of sacral erosion or paravertebral round shadows [1]. Lumbar spine MRI is valuable in diagnosing TCs, as TCs show long T1 and T2 signals, which are the same as cerebrospinal fluid, and can thus be distinguished from nerve sheath tumors [3, 13]. MRI not only demonstrates the location, size and shape of the cyst, but also directly measures whether it contains nerves and whether it is connected to the subarachnoid space, providing sufficient preoperative information for surgery [3, 14, 15]. Despite MRI being an effective method to diagnose TCs, as most TCs are asymptomatic, it can be difficult for doctors to determine if the symptoms are caused by a TC.

At present, TCs are easily overlooked and misdiagnosed. It is suggested that clinicians should increase their understanding of TCs, as well as assess all diagnostic details and provide timely auxiliary imaging examination and a careful differential diagnosis.

## Data Availability

The original materials in the report are available from the corresponding author on reasonable request.

## Abbreviations

TC: Tarlov cyst
